# Improved functionality, health related quality of life and decreased burden of disease in patients with ADHD treated with OROS^® ^MPH: is treatment response different between children and adolescents?

**DOI:** 10.1186/1753-2000-5-26

**Published:** 2011-07-26

**Authors:** Michael Berek, Andreas Kordon, Ludger Hargarter, Fritz Mattejat, Lara Slawik, Klaus Rettig, Barbara Schäuble

**Affiliations:** 1 Praxis für Kinder- und Jugendpsychiatrie, Eichenweg 73, D-30659 Hannover, Germany; 2 Klinik für Psychiatrie und Psychotherapie, Ratzeburger Allee 160, D-23538 Lübeck, Germany; 3 Janssen-Cilag Medical Affairs EMEA, Johnson & Johnson Platz 5a, D-41470 Neuss, Germany; 4 Klinik für Kinder- und Jugendpsychiatrie und -psychotherapie am Universitätsklinikum Gießen und Marburg, Standort Marburg, Hans-Sachs-Straße 4-6, D-35033 Marburg, Germany; 5 G.E.M. Gesellschaft für Evaluation und Qualitätssicherung in der Medizin mbH, Meerbuscher Str. 47, D-40670 Meerbusch, Germany

## Abstract

**Background:**

To compare clinical and health-related quality of life (HRQoL) outcomes between children and adolescents with ADHD treated with OROS^® ^MPH, using data from two large similarly-designed multicenter, prospective, open-label, single-arm, non-interventional studies.

**Methods:**

Pooled analysis (42603ATT4037, 42603 - ATT - 4001) including patients (6 to 18 years) with a confirmed diagnosis of ADHD. Patients were treated with OROS^® ^MPH for 12 weeks; ADHD symptoms, functioning, HRQoL, safety and tolerability parameters were assessed.

**Results:**

822 patients (583 children [6-12 years], 239 adolescents [13-18 years]) were included in the pooled analysis. Mean daily OROS^® ^MPH starting doses in the child and adolescent subgroups were 29.0 ± 11.7 and 37.6 ± 15.6 mg, respectively (p < 0.001). At study end (week 12), the overall mean daily dose was 35.5 ± 14.0 mg, with children and adolescents receiving 32.8 ± 12.7 and 42.0 ± 15.1 mg/day, respectively (p < 0.001). Significant (p < 0.0001: overall population, children, adolescents) symptomatic, functional and HRQoL improvements were observed from baseline to study end using the Conners' Parents Rating Scale (overall: 29.2 ± 10.7 [baseline] to 19.3 ± 11.3 [endpoint]), Children's Global Assessment Scale (overall: 58.5 ± 14.5 [baseline] to 69.6 ± 16.1 [endpoint]), and ILC-LQ0-28. At week 12, between-age group differences were seen in the individual ILC-LQ0-28 parameters: school performance (p = 0.001 [parents' assessment], p = 0.032 [childrens' assessment]), global QoL (p = 0.012 [parents']) and interests and hobbies (p = 0.023 [childrens']). Treating physician's planned continued use of OROS^® ^MPH in 76.9%, 86.0% and 79.3% of children, adolescents and the total population, respectively, at study end (p = 0.029 between-age subgroups). 195 of 822 patients (23.7%) experienced at least one treatment-emergent adverse event; most commonly reported AEs in the total group (≥4%) were insomnia (7.2%), anorexia (4.3%) and involuntary muscle contractions (4.1%). No clinically relevant changes in body weight or vital signs were observed.

**Conclusions:**

Clinically relevant differences between children and adolescents with ADHD are present. Adolescents appeared to have a lower health related quality of life and functioning compared to children at baseline, however, they were able to reach comparable ratings at endpoint for most items. Similarly, burden of disease decreased in patients and their carers. OROS MPH was generally safe and well tolerated.

## Background

The effects of ADHD in children are well-documented, impacting negatively on the child, peer group interaction, immediate family and home life as well as on the child's educational performance at school [1,2]. Children with ADHD often require special school education support services to aid their impaired learning [2,3].

Whilst an age-related decline in ADHD symptoms occurs throughout childhood [4], it is evident that ADHD persists into older age in the majority of individuals where it is associated with a range of clinical and psychosocial impairments [5]. Numerous follow-up studies of children with ADHD show that the disorder persists during adolescence and adulthood in around two-thirds of individuals with persistence of symptoms associated with continued clinical and psychosocial impairments [5]. A detailed longitudinal study of remission in boys with ADHD showed that syndromatic remission occurred in 60%, although most continued to experience ADHD symptoms (particularly inattention) and dysfunction after the age of 20 years [4]. Compared with healthy adolescents, fewer adolescents with ADHD enroll in college [2] and significantly higher absenteeism rates are observed [3]. Combined with continued learning disabilities, adolescents with ADHD also demonstrate impaired interpersonal relationships at school/college and at home, have significantly fewer close friends, more problems maintaining friendships, increased antisocial behavioural problems, greater parent-child conflict and parental hostility, and considerable overall negative impact as they progress into adulthood [1,2].

Whilst there may be differences between individuals in some ADHD domains, the continuing overall impact of childhood ADHD through adolescence appears to affect both genders to a similar extent. Owens et al. [6] recently demonstrated that very few girls diagnosed with childhood ADHD showed positive adjustment across multiple domains during adolescence, and concluded that the negative consequences of childhood ADHD for adolescent girls were equivalent to those reported in a separate, primarily male ADHD population [7].

Methylphenidate (MPH) is a well-established and recognized first-line stimulant treatment for children and adolescents with ADHD, decreasing symptom frequency and/or severity and improving functioning [8-10]. Immediate-release (IR) and extended-release (ER) MPH preparations are available, but these short-acting formulations have a number of potential limitations, including inconvenient multiple daily dosing that requires in-school/college administration and associated social attitudes and pressures, storage and handling problems [11], potential misuse and non-adherence leading to suboptimal treatment efficacy [12]. Osmotic, controlled-release (OROS) MPH, a long-acting MPH formulation, uses OROS^® ^(osmotic release oral system) technology to produce an ascending MPH plasma profile [13]. In clinical trials, once-daily OROS MPH has been shown to produce an extended duration of ADHD symptom control, consistent with an up to 12-hour duration of action [14-16].

The impact of health-related quality of life (HRQoL) is well-established [17,18] and it has been noted that HRQoL is not only lower in children and adolescents with ADHD when compared to healthy age- and sex-matched controls, but even when compared to children with other chronic diseases, including asthma [19]. However, there are currently limited data on HRQoL, everday functioning and well-being in children/adolescents with ADHD [17,20] and even less information documenting 'real world' changes in these parameters in patients with ADHD treated with MPH, or switching to OROS MPH.

This pooled analysis of two similarly-designed multicenter, prospective, open-label, single-arm, non-interventional studies [8-10], primarily explores differences with regard to effectiveness, tolerability and changes in HRQoL of OROS MPH between children and adolescents with ADHD [ICD-10 criteria (hyperkinetic disorders)] in a large cohort.

## Methods

### Study design and participants

This pooled analysis combines data from two similarly-designed large multicenter, prospective, open-label, single-arm, non-interventional studies (the LeCO study [8] and the GER-CON-2 study [9,10], 42603ATT4037, 42603 - ATT - 4001) which explored the efficacy, safety, tolerability and HRQoL outcomes of children and adolescents with ADHD treated with individualised dosing of OROS^® ^MPH (Concerta^®^; Janssen Cilag GmbH, Germany) over a 12-week treatment period. Patients had been treated with either atomoxetine, extended-release (ER) methylphenidate (GER-CON-2), or any ADHD-relevant psychostimulant (LeCO), before they started on OROS MPH. The pooled analysis specifically evaluated data in distinct age subgroups in order to explore potential differences in outcomes between children (aged 6-12 years) and adolescents (aged 13-18 years) with ADHD.

The two studies were conducted in paediatric, paediatric neurology, child and adolescent medicine practices or by child and adolescent psychiatrists. OROS^® ^MPH was prescribed according to its summary of product characteristics (SmPC). Starting and final dosages, as well as titration rates, were based on therapeutic effectiveness. Each study comprised five visits: baseline (week 0), brief follow-up visits after 1, 3 and 6 weeks of OROS^® ^MPH treatment, as well as a final visit after 12 weeks, or upon premature termination (study end).

Children and adolescents aged 6-18 years who had a confirmed diagnosis of ADHD (any subtype) by ICD-10 (F90.x: hyperkinetic disorders or F98.8) criteria, and in whom treatment with OROS^® ^MPH was medically indicated and planned by the treating physician, were eligible to participate in the studies. In the GER-CON-2 study, patients should have been pretreated with atomoxetine (Strattera^®^) or ER methylphenidate (Medikinet^® ^retard); in the LeCO study, patients should have been pretreated with any ADHD-relevant psychostimulant. Given the non-interventional design of the studies, there were no specific exclusion criteria.

### Ethics

An independent ethics committee (Freiburger Ethik-Kommission GmbH international [feki], Freiburg, Germany) reviewed and approved the two clinical study protocols, and consent was obtained from all participants and/or their care givers for data collection and source data verification.

### Symptomatic outcome measures

Symptomatic outcomes were assessed at week 0 (baseline; at the start of OROS^® ^MPH treatment), week 6 and week 12 (or upon termination for individuals who did not complete the study) using the Conners' Parent Rating Scale (CPRS) [21-23] which assesses symptoms of ADHD and other psychopathology and problem behaviour in children/adolescents aged 3-17 years. The scale uses a 4-point Likert format (0 = never, rarely; 1 = sometimes; 2 = frequently; 3 = very frequently and regularly). The two studies employed a short-form 18-item test with a total sum score ranging between 0 (best) to 54 (worst). A primary response was defined as a reduction in the total score of ≥30% and a secondary response as a reduction in score of ≥ 20%. Parents were asked to consider the patient's behaviour during the previous month.

### Health-related quality of life and functionality measures

Functionality and HRQoL outcomes were assessed at baseline and study end using the disease non-specific, 'Inventory for Assessment of Quality of Life in Children and Adolescents' (ILC [24-28]) which is a short questionnaire that takes approximately 5-15 minutes to complete. The German version of the ILC which was used in the two studies has been validated by Mattejat and Remschmidt [29]. After two independent forward, and one backward, translations, a Norwegian version of the ILC was investigated by Jozefiak and colleagues [28] in a Norwegian sample of 1997 school children aged 8-16 years and their parents. The ILC measures HRQoL over the past week and is sensitive to therapeutic interventions and changes in well-being over time.

Items 1-7: There are 7 core items of the ILC for normal children and patients, respectively, and their parents, consisting of: (1) school performance, (2) family functioning, (3) social integration, (4) interests and hobbies, (5) physical health, (6) emotional and physical well-being, and (7) global HRQoL ("overall").

Items 8-9: There are 2 additional items for the patients and their parents/caregivers: (8) problems (burden of present disorder/disease) and (9) overall evaluation of therapy (burden associated with the overall evaluation/diagnostic procedures and therapy).

Items 10-11: Additionally, there are 2 items for parents/caregivers of patients only: (10) problems (burden of present disorder/disease for parents/caregivers) and (11) overall evaluation of therapy (burden associated with the overall evaluation/diagnostic procedures and therapy for parents/caregivers).

All items on the ILC are rated on 5-point Likert scales (items 1-7: 1 = very good, 2 = rather good, 3 = mixed, 4 = rather bad, 5 = very bad; items 8-11: 1 = no problem, 2 = minor problem, 3 = moderate problem, 4 = significant problem, 5 = very significant problem). For children aged 6-11 years, the ILC is administered in a structured interview; adolescents and parents/caregivers complete the questionnaire on their own.

Three scores can be calculated from the 7 core items (items 1-7). For the purpose of this analysis, the overall score termed 'LQ0-28' was calculated if at least 4 of the 7 core items were answered. The LQ0-28 score ranges from 0 (worst) to 28 (best) and is calculated as LQ0-28: = ROUND[ABS(S*7/N-35)] where N and S are the number and sum of answered items, respectively, and ABS and ROUND the absolute and rounding function. Thus, scoring all 7 core items with 1 = 'very good' results in a LQ0-28 score of ROUND[ABS(7*1*7/7-35)] = ROUND[ABS(7-35)] = ROUND[ABS(-28)] = 28, scoring all 7 core items with 5 = very bad results in a LQ0-28 score of ROUND[ABS(7*5*7/7-35)] = ROUND[ABS(0)] = 0.

In the absence of a control group (healthy or placebo) in the two studies involved in this pooled analysis, data from Professor Mattejat's ILC-validation samples [29] were used for comparison: (1) data from 9418 ILC questionnaires completed by healthy children/adolescents and (2) data from 1140 ILC questionnaires completed by the parents of other healthy youngsters. From the validation samples, two samples matching the gender and age of the study group discussed in this paper were drawn. Validation sample data are based on a one-time evaluation.

The **Children's Global Assessment Scale **(C-GAS) is an instrument developed by Shaffer and colleagues to provide a global measure of the level of functioning in children and adolescents [30]. The scale provides a single global rating on a scale of 0 (worst) to 100 (best). The C-GAS was employed by the treating physician, based on information from an interview with the parents, at baseline (week 0) and weeks 6 and 12.

### Other assessments

Problems concerning social interactions and tasks were assessed via several non-validated questions. At each of the study visits, problems occurring in late afternoon (4 pm to 8 pm) that related specifically to 'playing with other children', 'household chores', 'school homework', 'going to bed', and 'behaviour towards visitors/at visits' were rated ('Please indicate whether the problems occurred between 4 pm and 8 pm and, if they occurred, how pronounced were they?) using a 4-point scale with the categories being: 0 = none, 1 = mild, 2 = moderate and 3 = severe.

Sleep quality ('How would you rate the sleep quality of patients in the last week?') and appetite ('How would you rate the appetite of patients in the last week?) were also assessed at each of the study visits, using a 5-point rating scale with the categories being: 'very good', 'good', 'satisfactory', 'sufficient', and 'insufficient'.

### Safety and tolerability assessments

Tolerability parameters included documentation of adverse events (AEs) throughout the two studies, recording of vital signs (blood pressure and heart rate) at all visits, and body weight at baseline and at the final visit.

### Data management and statistical analysis

All data were documented in Case Record Forms by the treating physician, entered into the database using a double data entry system and then checked for consistency and completeness. AEs were coded according to WHO Adverse Reaction Terminology.

Descriptive statistical estimators such as frequency counts, arithmetic means ± SD (standard deviation), median and range were used depending on the scale level. Pre-post comparisons were performed using Wilcoxon's test for dependent samples. Differences between children and adolescents, the primary topic of this paper, were analyzed by means of the Chi^2^- or the Mann-Whitney-U-test, respectively.

All tests were performed in a 2-sided manner in an exploratory sense without adjustment for multiple testing.

The evaluations were performed according to the intention-to-treat (ITT) principle. All enrolled patients who had received at least one dose of OROS^® ^MPH and who had at least one follow-up effectiveness assessment were available for the ITT-analysis of effectiveness data. Effectiveness data were presented as changes from baseline, missing values were imputed by the LOCF-method, where appropriate. The safety group included all patients who had at least one dose of OROS^® ^MPH and had safety data reported.

## Results

### Baseline demographics and disease characteristics

This pooled analysis evaluated data from a total of 822 patients with ADHD; 598 patients were from the LeCO study [8] and 224 patients were from the GER-CON-2 study [9,10]. Relevant baseline patient demographics and disease characteristics are shown in Table 1. Of the 822 patients in the total ITT and safety analysis, there were 583 children (mean age 9.8 ± 1.6 years, range 6-12 years) and 239 adolescents (mean age 14.4 ± 1.3 years, range 13-18 years), with 85% of all participants being male. The overall mean age at first diagnosis of ADHD was 8.1 ± 2.5 years and the average duration since diagnosis at study start was 2.4 ± 1.6 and 4.3 ± 2.7 years in the children and adolescent subgroups, respectively (between-group difference, p < 0.001). The mean duration of observation was 86.7 ± 28.5 days in the overall ITT population. Approximately two-thirds of all patients had a diagnosis of F90.0 (disturbance of activity and attention).

**Table 1:** Demographic data and disease characteristics (by age group).

Age	6-12 years N = 583	13-18 years N = 239	All patients N = 822	**U-test**^**1**^ **Chi**^**2**^**-test**^**2**^
Gender, n (%)				p = 0.460^2^

Female	84 (14.41)	40 (16.74)	124 (15.09)	

Male	499 (85.59)	199 (83.26)	698 (84.91)	

Age at study start (years)				

mean ± SD	9.75 ± 1.59	14.38 ± 1.29	11.10 ± 2.58	p < 0.001^1^

minimum, median, maximum	6,10,12	13,14,18	6,11,18	

Age at study start (years) by group, n (%)				

6-9	251 (43.05)	0 (0.00)	251 (30.54)	

10-12	332 (56.95)	0 (0.00)	332 (40.39)	

13-15	0 (0.00)	197 (82.43)	197 (23.97)	

16-18	0 (0.00)	42 (17.57)	42 (5.11)	

Age at first diagnosis of disease (years)				p < 0.001^1^

valid N	565	220	785	

mean ± SD	7.31 ± 1.85	9.97 ± 2.86	8.06 ± 2.49	

minimum, median, maximum	2,7,12	1.0,10.0,16.0	1.0,8.0,16.0	

Duration of disease at study start (years)				p < 0.001^1^

valid N	565	220	785	

mean ± SD	2.41 ± 1.56	4.27 ± 2.71	2.93 ± 2.12	

minimum, median, maximum	0.03,2.1,7.9	0.17,3.8,12	0.03,2.51, 12	

Diagnosis of ADHD (ICD-10) n (%)^3^				

F90.0: disturbance of activity and attention	364 (62.44)	155 (64.85)	519 (63.14)	p = 0.567^2^

F90.1: hyperkinetic conduct disorder	227 (38.94)	89 (37.24)	316 (38.44)	p = 0.707^2^

F90.8: other hyperkinetic disorder	8 (1.37)	6 (2.51)	14 (1.70)	p = 0.396^2^

F90.9: hyperkinetic disorder, unspecified	18 (3.09)	3 (1.26)	21 (2.55)	p = 0.205^2^

others	41 (7.03)	24 (10.04)	65 (7.91)	p = 0.287^2^

Previous and/or concomitant diseases (ICD-10) n (%)^3^				

No	351 (60.21)	145 (60.67)	496 (60.34)	p = 0.964^2^

Others	47 (8.06)	26 (10.87)	73 (8.88)	

F91.X: conduct disorder	134 (22.98)	53 (22.18)	187 (22.75)	p = 0.288^2^

F91: incl. F91.3 oppositional defiant disorder	103 (17.67)	37 (15.48)	140 (17.03)	p = 0.512^2^

F41: anxiety disorder	25 (4.29)	7 (2.93)	32 (3.89)	p = 0.534^2^

F42: obsessive-compulsive disorder	12 (2.06)	3 (1.26)	15 (1.82)	p = 0.599^2^

F1X: substance abuse	0 (0.00)	6 (2.51)	6 (0.73)	p = 0.001^2^

1,2 p-values from the corresponding test between age groups: 6-12 years and 13-18 years

3 multiple responses possible; Chi^2^-test always yes vs. no

### Treatment with OROS MPH

Patients had been treated with atomoxetine, ER MPH or IR MPH prior to study start. The mean daily starting dose of OROS MPH was 29.0 ± 11.7 (median: 36 mg, range: 18-72 mg) [children] and 37.6 ± 15.6 mg (median: 36 mg, range: 18 to 72 mg) [adolescents], respectively (p < 0.001). At study end, the mean OROS MPH daily dose had increased significantly (p < 0.001) to 32.8 ± 12.7 and 42.0 ± 15.1 mg for children and adolescents, respectively, with no between-group difference (p = 0.579) [Table 2]. Similarly, mean daily OROS MPH doses, expressed in mg/kg bodyweight, were 0.9 ± 0.4 (median: 0.82 mg/kg/day) [children] and 0.7 ± 0.4 (median 0.66 mg/kg/day) [adolescents], respectively (p < 0.001). At study end, the mean OROS MPH daily dose (by bodyweight) had increased significantly (p < 0.001) for both children and adolescents from baseline, with no between-group difference (p = 0.665) [Table 2].

**Table 2:** Details of pre- and study medication.

Age	6-12 years N = 583	13-18 years N = 239	All patients N = 822	**U-test**^**2**^ **Chi**^**2**^**-test**^**3**^
**Reason for starting OROS MPH, n (%)**^1^				

N	142 (100.00)	82 (100.00)	224 (100.00)	p < 0.090^3^

insufficient effectiveness	92 (64.79)	63 (76.83)	155 (69.20)	

adverse events	5 (3.52)	4 (4.88)	9 (4.02)	

combination of both	45 (31.69)	15 (18.29)	60 (26.79)	

**Dose of OROS MPH**				

starting dose (mg/day)	29.05 ± 11.71	37.58 ± 15.62	31.53 ± 13.52	p < 0.001^2^

minimum, median, maximum	18, 36, 72	18, 36, 108	18, 36, 108	

last visit	32.82 ± 12.68	41.95 ± 15.07	35.47 ± 14.04	p < 0.001^2^

minimum, median, maximum	18, 36, 72	18, 36, 108	18, 36, 108	

Difference (mean ± SD)	3.77 ± 9.03	4.37 ± 10.43	3.94 ± 9.46	p = 0.579^2^

Wilcoxon p-value	< 0.0001	< 0.0001	< 0.0001	

**Dose of OROS MPH (mg/day/kg BW)**	**N = 527**^**4**^	**N = 223^4^**	**N = 750^4^**	

starting dose (mg/day/kg BW)	0.90 ± 0.41	0.73 ± 0.39	0.85 ± 0.41	p < 0.001^2^

minimum, median, maximum	0.30,0.82,3.13	0.20,0.66,2.77	0.20,0.78,3.13	

last visit	1.03 ± 0.45	0.82 ± 0.37	0.97 ± 0.44	p < 0.001^2^

minimum, median, maximum	0.29,0.95,3.13	0.20,0.72,2.32	0.20,0.88,3.13	

Difference (mean ± SD)	0.13 ± 0.32	0.08 ± 0.20	0.11 ± 0.29	p = 0.665^2^

Wilcoxon p-value	< 0.0001	< 0.0001	< 0.0001	

**Days of treatment with OROS MPH**	82.90 ± 30.32	87.36 ± 27.15	84.20 ± 29.49	p = 0.012^2^

**Days of observation**	85.97 ± 29.35	89.05 ± 26.10	86.87 ± 28.46	p = 0.046^2^

**Number (%) of patients with at least one additional application of IR MPH between study start and study end**				

yes	180 (30.87)	68 (28.45)	248 (30.17)	p = 0.546^3^

no	403 (69.13)	171 (71.55)	574 (69.83)	

1 Data only available for patients (n = 224) from the GER-CON-2 study

2,3 p-values from the corresponding test between age groups: 6-12 years and 13-18 years

4 Determined only in patients with bodyweight documented at first and last visit

Abbreviations: **BW **= bodyweight; **IR **= immediate-release; **MPH **= methylphenidate.

Mean OROS MPH treatment duration was 83.0 ± 30.3 days (children) and 87.4 ± 27.2 days (adolescents) (between-group difference, p = 0.012).

### Conners' Parent Rating Scale

**Figure 1:**
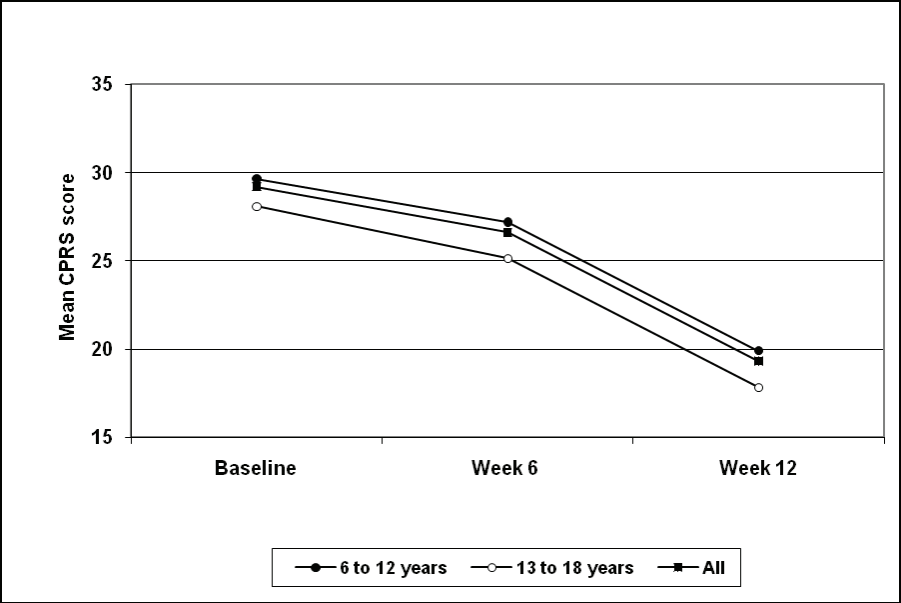
**Mean Conners' Parent Rating Scale (CPRS) scores: overall (n = 822) and age subgroups**. Data are presented for the intention-to-treat analysis, last observation carried forward. Lower scores denote improvement. Baseline to week 12 improvements were p < 0.0001 for all groups (Wilcoxon test). Assessments at week 6 are only based on data from 224 patients.

The overall mean CPRS score improved from 29.2 ± 10.7 at baseline to 19.3 ± 11.3 at week 12 (endpoint) [p < 0.0001; Figure 1]. Adolescents had slightly, but not statistically significantly, better CPRS scores than children (Figure 1). Significant improvements between baseline and week 12 (endpoint) were seen in children (baseline: 29.7 ± 10.8, week 12: 19.9 ± 11.6, p < 0.0001) and adolescents (baseline: 28.1 ± 10.1, week 12: 17.8 ± 10.4, p < 0.0001). However, improvements were not significantly different between age groups (p = 0.279).

### Children's Global Assessment Scale

**Figure 2:**
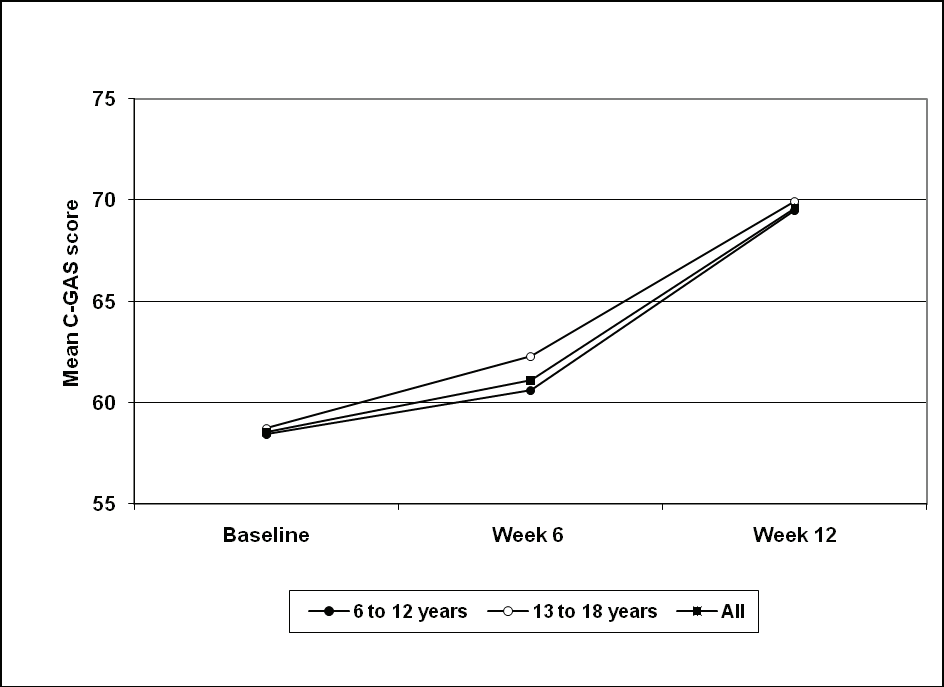
**Mean Children's Global Assessment Scale (C-GAS) scores**. Data are presented for the overall population (n = 822) and by age subgroups (intention-to-treat, last observation carried forward). Higher scores denote improvement. Baseline to week 12 improvements were p < 0.0001 for all groups (Wilcoxon test). Assessments at week 6 are only based on data from 224 patients.

The mean C-GAS score for all patients improved from 58.5 ± 14.5 at baseline to 69.6 ± 16.1 at week 12 (p < 0.0001). As shown in Figure 2, significant improvements between baseline and week 12 were recorded for children (11.0 ± 14.1; p < 0.0001) and adolescents (11.2 ± 13.3; p < 0.0001), respectively. Improvements were not significantly different between age groups (p = 0.402).

### Inventory for assessing health-related quality of life (ILC)

**Figure 3:**
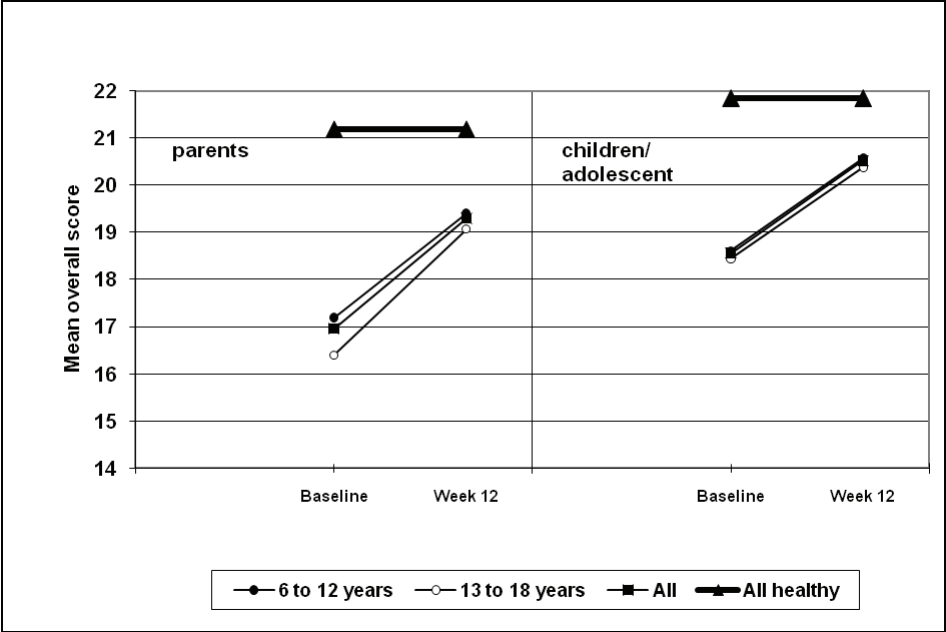
**Mean overall improvements in health-related quality of life (ILC-LQ0-28)**. Data are presented for the overall population and by age subgroups (intention-to-treat, last observation carried forward). High scores denote high quality of life.

The mean ILC LQ0-28 score for children improved significantly from 17.2 ± 3.9 at baseline to 19.4 ± 4.0 at endpoint (p < 0.0001) according to parents' ratings, and from 18.6 ± 4.1 to 20.6 ± 3.9 (p < 0.0001) according to patients' ratings. For adolescents, the mean ILC LQ0-28 score improved significantly from 16.4 ± 3.9 at baseline to 19.1 ± 4.0 at study end (p < 0.0001) according to parents' ratings, and from 18.4 ± 3.7 to 20.4 ± 3.6 (p < 0.0001) according to patients' ratings (Figure 3). Mean baseline ILC LQ0-28 scores were significantly (p = 0.009) lower in the adolescent subgroup for parents' ratings although between-group differences at week 12 were not significantly different.

**Figure 4:**
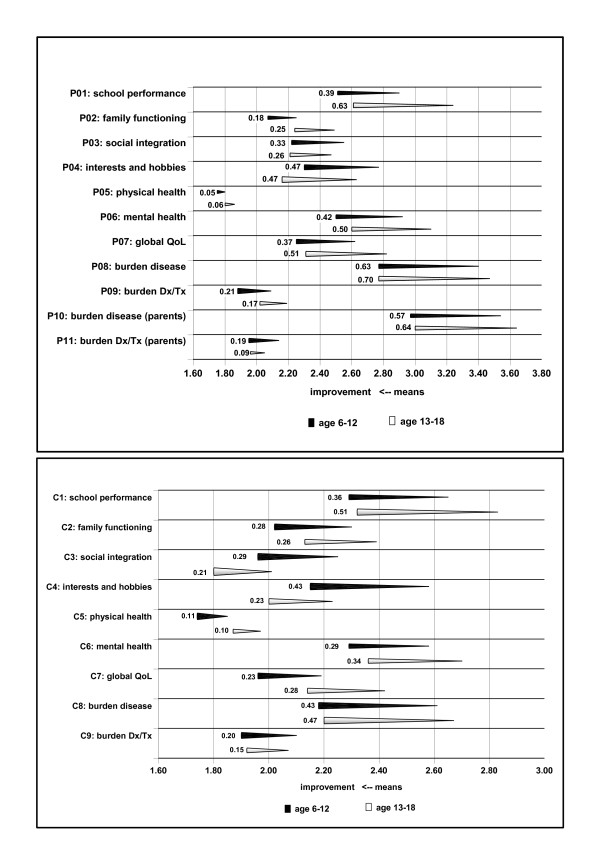
**Individual health-related quality of life (ILC) item scores assessed by (a) parents (P), and (b) patients (children/adolescents [C]). **Mean scores at baseline and at study end (week 12) [intention-to-treat, last observation carried forward]. Dx = diagnostic procedures. Tx = therapeutic procedures. The right sides of the bars represent mean baseline values, the left sides mean values at week 12, the numbers mean improvements between both time points.

Mean baseline and study end (week 12) scores for each individual ILC item are shown in Figure 4a (parents' assessments) and Figure 4b (patients' assessments), for the overall population and the 'children' and 'adolescents' subgroups. At baseline, parents' assessments showed significant differences between children and adolescents in family functioning (p = 0.001), mental health (p = 0.006) and global QoL (p = 0.002). Patients' assessments showed significant between-age group differences at baseline in school performance (p = 0.008), social integration (p = 0.006) and physical health (p = 0.014).

At study end, significant improvements from baseline were observed in the overall patient population for all individual items according to parents' ratings (p = 0.03 for physical health and p < 0.001 for all other items). Analysis of both age subgroups showed that improvements in the 'physical health' item were not statistically significant according to parents' ratings (p = 0.12); all other parameters were significantly improved (Figure 4a). According to patients' ratings, all individual ILC items improved significantly in both age subgroups and in the overall population (Figure 4b). At study end, between-age group differences were seen in school performance (p = 0.001 [parents' assessment], p = 0.032 [patients' assessment]), global QoL (p = 0.012 [parents']) and interests and hobbies (p = 0.023 [patients']).

The additional ILC item regarding the patient's burden associated with ADHD improved on average by 0.44 ± 1.11 (p < 0.0001) in the overall population when assessed by the patients and by 0.65 ± 1.10 (p < 0.0001) when assessed by the parents, with similar improvements in both age subgroups. For patients and parents, this was the individual item with the largest mean improvement. On average, the parents' burden of disease improved from baseline to study end by 0.59 ± 1.03 (p < 0.0001) in the overall population. The burden associated with diagnostic or therapeutic procedures also improved significantly for parents and patients in the overall population (p < 0.001).

The two individual ILC items with the worst baseline scores in the analysis improved largely: item 1 (school performance) and item 6 (mental health). These items were only slightly worse or comparable to those of healthy age-matched controls.

### Other assessments

**Figure 5:**
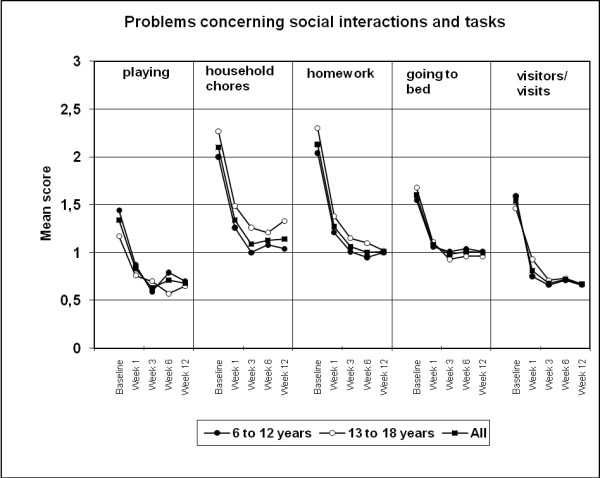
**Mean scores for problems concerning social interactions and tasks occurring in late afternoon (4 pm to 8 pm)**. Data presented for the overall population (n = 224) and children and adolescent subgroups (intention-to treat, last observation carried forward). At week 12, all improvements from baseline were significant (p ≤ 0.0001).

Based on data from 224 patients, at week 12, children and adolescents showed significant (p ≤ 0.0001) improvements from baseline in problems concerning social interactions and tasks occurring in late afternoon (4 pm to 8 pm) [Figure 5]. With the exception of a significant baseline difference between children and adolescents in the mean score for 'household chores' (p = 0.036) [no between-group difference at week 12 for this parameter], no significant between-age group differences were observed for any of the social interaction parameters.

In the overall population (n = 822), quality of sleep (p = 0.0034) and appetite (p = 0.0109) improved significantly from baseline to study end. Sleep quality also improved significantly from baseline in the adolescent population (p = 0.0143). Significant between-age group differences in sleep quality were observed at baseline (p = 0.04) and study end (p = 0.002).

Overall, quality of sleep and appetite improved in 23.7% and 25.9% of patients, respectively, and worsened in 32.5% and 32.4% of patients, respectively.

Continued use of OROS MPH at study end by the treating physician was planned in 76.9%, 86.0% and 79.3% of children, adolescents and the total population, respectively. The difference between age subgroups (children versus adolescents) was significant (p = 0.029).

### Tolerability

In the overall population, 195 of the 822 patients (23.7%) reported experiencing at least one AE causally related to treatment (at least possible) during the study. No significant difference (p = 0.066) in the incidence of AEs were observed in children (25.6%) and adolescents (19.3%). Overall, the study was prematurely terminated due to AEs by 60 patients (7.3%), with 8.2% of children and 5.0% of adolescents terminating the study due to AEs (p = 0.144). The incidences of the most common treatment-related AEs, and AEs as the reason for study termination, are presented in Tables 3 and 4.

**Table 3:** Adverse events causally related to treatment (at least possible) reported in at least 2% in any subgroup of patients (sorted by percentages in the 'All patients' group).

Age	6-12 years N = 583		13-18 years N = 239		All patients N = 822	
**Adverse event (AE)** **[preferred term]**	**n**	**%**	**n**	**%**	**n**	**%**

Patients with at least one AE*	149	25.56	46	19.25	195	23.72

Insomnia	48	8.23	11	4.60	59	7.18

Anorexia	31	5.32	4	1.67	35	4.26

Muscle contractions involuntary	29	4.97	5	2.09	34	4.14

Medicine ineffective	13	2.23	5	2.09	18	2.19

Nervousness	13	2.23	4	1.67	17	2.07

Headache	10	1.72	5	2.09	15	1.82

Concentration impaired	7	1.20	7	2.93	14	1.70

Aggressive reaction	8	1.37	5	2.09	13	1.58

Abdominal pain	12	2.06	0	0.00	12	1.46

Weight decrease	4	0.69	6	2.51	10	1.22

* Chi2- Test: p = 0.066; some patients had more than one AE.

**Table 4:** Adverse events as reason for study termination reported in at least 1 patient in the total group (sorted by percentages in the total group).

Age	6-12 years N = 583		13-18 years N = 239		All patients N = 822	
**Adverse event (AE)** **[preferred term]**	**n**	**%**	**n**	**%**	**n**	**%**

Patients with at least one AE*	48	8.23	12	5.02	60	7.30

Insomnia	16	2.74	3	1.26	19	2.31

Anorexia	6	1.03	2	0.84	8	0.97

Emotional lability	4	0.69	3	1.26	7	0.85

Muscle contractions involuntary	6	1.03	1	0.42	7	0.85

Depression	3	0.51	3	1.26	6	0.73

Abdominal pain	5	0.86	0	0.00	5	0.61

Aggressive reaction	4	0.69	1	0.42	5	0.61

Nervousness	4	0.69	1	0.42	5	0.61

Headache	1	0.17	3	1.26	4	0.49

Nausea	3	0.51	1	0.42	4	0.49

Apathy	1	0.17	2	0.84	3	0.36

Tachycardia	2	0.34	1	0.42	3	0.36

Anxiety	2	0.34	0	0.00	2	0.24

Concentration impaired	1	0.17	1	0.42	2	0.24

Dysphagia	2	0.34	0	0.00	2	0.24

Weight decrease	1	0.17	1	0.42	2	0.24

* Chi2- Test: p = 0.144; some patients had more than one AE.

No serious AEs (SAEs; any adverse experience that resulted in any of the following outcomes: death, a life-threatening experience, inpatient hospitalization or prolongation of existing hospitalization [except inpatient rehabilitation and inpatient hospitalisations planned prior to the study], a persistent or significant disability/incapacity, or a congenital anomaly/birth defect) occurred that were considered to be causally related to OROS^® ^MPH treatment.

Based on investigators' assessments, the three most commonly reported treatment-related AEs in the total population, children (6-12 years) and adolescents (13-18 years) were insomnia (7.2%, 8.2% and 4.6% of patients, respectively), anorexia (4.3%, 5.3% and 1.7%, respectively) and involuntary muscle contractions (tics) [4.1%, 5.0% and 2.1%, respectively] (Table 3). Insomnia (2.3%, 2.7% and 1.3% of patients, respectively) and anorexia (1.0%, 0.8% and 1.0%, respectively) were also the AEs most commonly leading to premature termination (Table 4) in the overall population, children and adolescents, respectively).

Based on data from 598 patients, the overall tolerability of OROS MPH was rated as 'very good' (37.8% and 34.8%) or 'good' (44.0% and 45.0%) by the majority of physicians and parents, respectively. There was no significant between-age group difference in the tolerability of OROS MPH as rated by physicians (p = 0.065), whereas the between-age group difference, as rated by parents, was significant (p = 0.004).

On average, no clinically relevant weight changes, or changes in vital signs, were observed during the study.

## Discussion

This pooled analysis of over 800 children and adolescents with ADHD shows that treatment with OROS MPH improved symptomatic, functional and HRQoL measures in a 'real world' setting. Significant improvements from baseline (e.g. the start of OROS MPH treatment) were observed with respect to symptoms, functioning and HRQoL across both age categories (children and adolescents) and for males and females, as reported by patients and parents at week 12. Whilst differences between the two age populations were not always statistically significant for many of the evaluated parameters, significant differences were observed in sleep quality and several HRQoL parameters. Notably, at study end, between-age group differences were seen in school performance (p = 0.001 [parents' assessment], p = 0.032 [patients' assessment]), global QoL (p = 0.012 [parents' assessment]) and interests and hobbies (p = 0.023 [patients' assessment]). With the exception of 'interests and hobbies' (patients' assessment), the greatest improvements in sleep quality and these HRQoL parameters were observed in adolescents. Given that ADHD persists beyond childhood into older age where it is associated with a range of clinical and psychosocial impairments [5], it is of interest that our observations indicate that treatment with OROS MPH is associated with even greater improvements in several HRQoL parameters in adolescents compared with children. Overall, similar findings were reported recently from a pooled analysis of five clinical trials in which atomoxetine was generally shown to be effective in improving certain aspects of HRQoL in 794 children and adolescents with ADHD [31].

In addition, in our study, all mean HRQoL values were close to those reported for healthy controls after 12 weeks, although differences were not always statistically significant. OROS MPH was well tolerated, exhibiting a safety profile in line with the SmPC.

Overall, the results from this 'real world' pooled analysis align with data from controlled clinical studies that have evaluated treatment of children/adolescents with ADHD with OROS MPH [14,32-34]. A 3-week UK and German multicenter, open-label study showed that children and adolescents (n = 105) with ADHD maintained (teacher ratings) or had improved (parent/patient ratings) symptom control after transitioning to OROS MPH from IR MPH. The authors suggested that the prolonged duration of action of OROS MPH improved symptom control beyond the structured school day and that increased improvements seen by parents/caregivers, resulting from improved symptom control in the after-school period, dominated the ratings [14]. Chou and colleagues [33] reported benefits in Taiwanese children with ADHD (n = 137) who switched to OROS MPH (for ≥ 3 weeks) after exhibiting consistently poor adherence to at least 3 months' treatment with IR MPH. Significant improvements were noted in behavioural symptoms and family/school measures. Improved neurocognitive performance was also reported in an open-label study involving Korean children (n = 102) with ADHD who were switched from IR MPH to OROS MPH for 28 days [34]. The clinical improvement with OROS MPH seen in these studies probably relates to improved compliance with a long-acting once-daily medication and more consistent serum levels of MPH achieved with the OROS formulation. Indeed, in a multicenter, double-blind, placebo-controlled crossover study, Sonuga-Barke and colleagues showed that differences in the pharmacokinetic profiles of OROS^® ^MPH (up to 12-hour duration of action) and a once-daily ER MPH formulation (Metadate^®^) resulted in predictably different efficacy profiles over 12 hours in a classroom setting of children with ADHD [32].

Further to clinical studies that have demonstrated the successful transition from IR or ER MPH to OROS MPH, several studies have shown the long-term benefits of stimulant medication in children with ADHD. In a population-based birth cohort, Barbaresi and colleagues [35] have shown that stimulant treatment of 370 children with ADHD improved long-term school outcomes, improving reading achievement, and decreasing school absenteeism and grade retention. A longitudinal case-control 10-year follow-up study recently provided new evidence that stimulant treatment of children/adolescents with ADHD may be protective against adverse educational outcomes [36]. In this study, treatment with stimulants significantly reduced the likelihood of disruptive behaviour and recipients were less likely to repeat a school/college grade than non-treated patients with ADHD. In our study, children and adolescents with ADHD were successfully treated with OROS MPH with improvements in symptomatic, functional and HRQoL measures; although patients were only observed for 3 months, physician's planned to continue longer-term use of OROS MPH in 77% and 86% of children and adolescents, respectively (p = 0.029 between-age subgroups). Furthermore, van den Ban and colleagues [37] have recently shown that the use of long-acting OROS MPH is associated with lower discontinuation rates in patients with ADHD in the Netherlands, compared with rates observed when only shorter-acting medications were previously available.

This pooled analysis is not without some limitations. Firstly, whilst the two contributing studies were of similar design, they were open-label and single arm, with no control group. The overall population was restricted to patients who were treated with OROS MPH and patient motivation for study participation could have been prompted by suboptimal efficacy, adverse events, and/or low HRQoL with previous management strategies, possibly contributing to a bias towards HRQoL improvement. However, given the selection criteria, the pooled analysis explores outcomes in a large and broad patient population by including patients seen in 'real world' daily practice who may not participate in randomized controlled trials. In addition, the relatively long study duration (12 weeks) may provide valuable information on longer term treatment outcomes.

In conclusion, treatment with OROS MPH for 12 weeks in children and adolescents with ADHD was well tolerated and was associated with improvements from baseline in symptoms, functioning and HRQoL. Adolescents appeared to have a lower health related quality of life and functioning compared to children at baseline, however, they were able to reach comparable ratings at endpoint for most items. Furthermore, both parents and affected individuals reported improved HRQoL and a relevant decrease in burden of disease.

## Competing interests

LH, LS and BS are employees of Janssen-Cilag, Germany. KR is a consultant working for GEM, Meerbusch, Germany, who was hired and paid by Janssen-Cilag to carry out the statistical analyses. MB, AK and FM declare no competing interests.

## Authors' contributions

All authors contributed equally to data analyses, interpretation, content development and critical input. All authors read and approved the final manuscript.
